# MicroRNA miR171b Positively Regulates Resistance to Huanglongbing of Citrus

**DOI:** 10.3390/ijms24065737

**Published:** 2023-03-17

**Authors:** Yuanda Lv, Yun Zhong, Bo Jiang, Huaxue Yan, Shuang Ren, Chunzhen Cheng

**Affiliations:** 1 Institute of Fruit Tree Research, Guangdong Academy of Agricultural Sciences, Guangzhou 510640, China; lvyuanda2008@163.com (Y.L.);; 2 Key Laboratory of South Subtropical Fruit Biology and Genetic Resource Utilization, Ministry of Agriculture and Rural Affairs, Guangzhou 510640, China; 3 Guangdong Provincial Key Laboratory of Tropical and Subtropical Fruit Tree Research, Guangzhou 510640, China

**Keywords:** miR171b, Huanglongbing, overexpression, genetic transformation

## Abstract

Huanglongbing (HLB) is one of the most severe citrus diseases in the world, causing huge economic losses. However, efficient methods of protecting citrus from HLB have not yet been developed. microRNA (miRNA)-mediated regulation of gene expression is a useful tool to control plant diseases, but the miRNAs involved in regulating resistance to HLB have not yet been identified. In this study, we found that miR171b positively regulated resistance to HLB in citrus. Upon infection with HLB bacteria, the bacteria were detected in the second month in the control plants. However, in the miR171b-overexpressing transgenic citrus plants, the bacteria could not be detected until the 24th month. RNA-seq data indicated that multiple pathways, such as photosynthesis, plant–pathogen interaction, the MAPK signaling pathway, etc., might be involved in improving the resistance to HLB in miR171b-overexpressing plants compared with the control. Finally, we determined that miR171b could target *SCARECROW-like* (*SCL*) genes to downregulate their expression, which then led to promoted resistance to HLB stress. Collectively, our results demonstrate that miR171b plays a positive regulatory role in resistance to citrus HLB, and provides a new insight into the role of miRNAs in the adaptation of citrus to HLB stress.

## 1. Introduction

Citrus Huanglongbing (HLB) disease has spread globally to the major citrus-producing areas, posing a serious threat to the sustainability of the world citrus industry [1]. The disease is caused by *Candidatus* Liberibacter asiaticus (*C*Las), *Candidatus* Liberibacter africanus (*C*Laf), and *Candidatus* Liberibacter americanus (*C*Lam), which are members of the α-subclass of prokaryotic Proteobacteria [2]. *C*Las is the most widespread and severe one [3]. These bacteria have highly simplified genomes that lack the key genes for autotrophy [4], and therefore exploit host cells to survive. Their natural hosts include citrus (subfamily Aurantioidae) and citrus relatives. To disperse, the bacteria must be vectored by insects, either the Asian citrus psyllid *Diaphorina citri* (ACP), which transmits *C*Las, or the African psyllid *Trioza erytreae*, which transmits *C*Laf [5]. Currently, the widely used strategies in the control of HLB include pathogen-free propagation materials, the timely eradication of diseased trees to reduce field inoculum, and the aggressive suppression of ACP populations [6,7]. It is very difficult to wipe out ACP from an area where the insect has established a population, and satisfactory control is not always achieved, even when using very aggressive control programs.

Breeding for disease resistance is the most cost-efficient and environmentally friendly way to curb the spread of diseases [8]. The conventional method of breeding for resistance is the incorporation of a disease-resistance gene into a plant genome through a cross followed by multiple back-crosses [9]. This strategy is largely dependent on the availability of usable resistance genes. Unfortunately, no HLB resistance gene has been identified in citrus or its close relatives *Fotunella* and *Poncirus* so far [10]; therefore, little progress has been made in breeding for HLB resistance. Nevertheless, some intergeneric hybrids between *Citrus* and *Poncirus* have shown some degrees of HLB tolerance and have been released as rootstocks [11,12,13,14]. Evidently, different and novel approaches should be tested, such as the use of host susceptibility genes (*S*-genes), which are essential to the survival of the invading pathogens [15]. Theoretically, HLB resistance can be achieved if dysfunctional *S*-genes are additively pyramided, if one is not enough.

There are several ways to interrupt gene function, including T-DNA insertion, CRISPR/Cas-mediated gene editing, and mRNA targeting, among others [16]. MicroRNAs (miRNA), a class of small (21–23 nt), single-stranded, non-coding RNA molecules, could bind target mRNA in a complementary base-pairing manner to induce the degradation of the target mRNA [17,18].

miRNAs are key players in regulating abiotic stress responses in plants. Yang et al. found that the expression of most miRNAs exhibits a negative correlation with the expression of their targets under salt stress in the comparative analysis of two sweet potato (*Ipomoea batatas* (L.) Lam.) cultivars with different salt sensitivity [19,20]. miR156, miR166, and miR171 regulate transcription factor genes to adapt maize (*Zea mays* L.) to low phosphorus stress [21]. miR166, miR171, miR390, miR156, and miR168 respond to Cd stress in rice (*Oryza sativa*) [22,23]. Plant biotic stress responses also involve miRNAs. For example, miR1507 regulates rhizobial symbiosis in legume species [24], and miR393, miR160, and miR167 were found to be upregulated in tomato leaves infected with *Pseudomonas syringae* [25]. Some cassava (*Manihot esculenta*) miRNAs, miR160, miR167, miR390, and miR393, respond to *Xanthomonas* infection, similar to those in *Arabidopsis* [26]. miRNAs have been evidenced to improve plant resistance but have not been used in citrus HLB.

A miRNA can target one or several species of mRNAs and vice versa [27]. This is exemplified by miR171. miR171 can target multiple members of the *SCARECROW-like* (*SCL*) transcription factor gene family [28,29]. The *Scarecrow-like 6* (*SCL6*) gene family is primarily involved in the response of tomato to GA/Auxin signals [30,31]. miR171 can thus regulate axillary bud growth, embryogenesis induction, and leaf and root activities that heavily depend on the regulation of plant growth hormones, as shown by its involvement in embryo genes in lily (*Lilium brownii var. viridulum*) [32]. Additionally, miR171 is associated with associated with biotic and abiotic stresses. For example, Arabidopsis and rice miR171 are involved, through interaction with miR169, in the salt stress response [29]. Soybean (*Glycine max* (L.) Merr.) miR171 enhances the colonization of arbuscular mycorrhizae in roots [33], while citrus miR171b, by joining with miR167h, participates in the symbiosis of arbuscular mycorrhizae in roots [34]. In ORS571 rhizobium infected wheat, the expression level of miR171 in buds was higher than that in roots to improve the resistance [35]. In citrus, miR171 and miR156 were involved in the stress of *Citrus psorosis virus* by down-regulating the target genes [36].

In our previous study, we analyzed the changes in miRNA expression in citrus roots infected with HLB. The results demonstrate that the differential expression of miRNAs mainly targeted genes involved in stress response, metabolism, transcription, growth and development. In addition, one of the most striking findings was that miR171b expression is significantly induced by HLB infection [37]. In this work, finding the mechanism of miR171b improved resistance to HLB, which is of great theoretical interest and has potential applications in breeding for disease resistance. We set out to determine whether the miR171b plays a role in the defense of citrus against *C*Las.

## 2. Results

### 2.1. Generation of Transgenic Plants Overexpressing pre-ctr-miR171b

**Figure 1:**
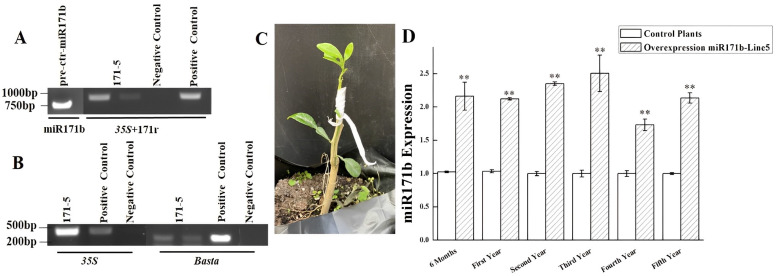
Generation of transgenic plant overexpression of pre-ctr-miR171b. (**A**). pre-ctr-miR171b cloning and the transgenic plant identification with *35S*F + 171r primers. (**B**). Confirm the 171-5 is the transgenic plant with *35S* and Basta. (**C**). Transgenic plants at two months post-grafting. (**D**). Quantitative real-time PCR results of miR171b in transgenic line 171-5. Experiments were repeated twice with similar results. ** indicate *p* values of <0.01, compared with the non-transgenic plants.

Sweet orange pre-ctr-miR171b was successfully cloned (Figure 1A) into a TA-cloning vector using a pair of 171f/171r primers. The insert was excised from the TA vector using double enzyme digestion and inserted into the pFGC5941 over-expression vector. Transgenic citrus plants were obtained using *Agrobacterium tumefaciens*-mediated transformation (Figure 1B). A total of 16 Basta-resistant transgenic buds were grafted onto rootstocks. DNA was extracted from the leaves of the transgenic lines when 5–8 true leaves were present, and the pre-ctr-miR171b transgene was detected via PCR. The transgenic plant was eventually identified and designated as 171-5 (Figure 1C).

Transgene expression in 171-5 was monitored via qPCR for five years. It was found that the expression of the gene was stable, varying from 1.6- to 2.5-fold, and averaged 2.16-fold compared with those in non-transgenic plants (Figure 1D).

### 2.2. The 171-5 Asexual Progenies Show HLB-Tolerance

**Figure 2:**
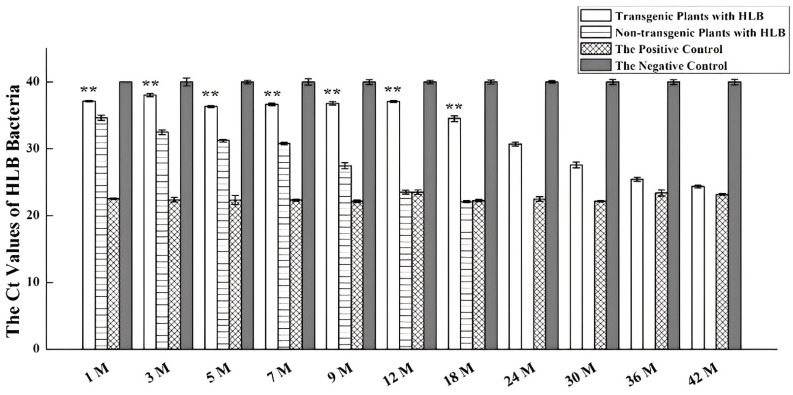
Quantitative real-time PCR detection of HLB in transgenic plants overexpressing miR171b. The horizontal axis represents detection time points following the inoculation of HLB. The vertical axis represents Ct values of the HLB-specific amplicon. Experiments were repeated twice with similar results. ** indicates *p* values of <0.01 compared with the non-transgenic plants. The M on the *X*-axis means the month.

**Figure 3:**
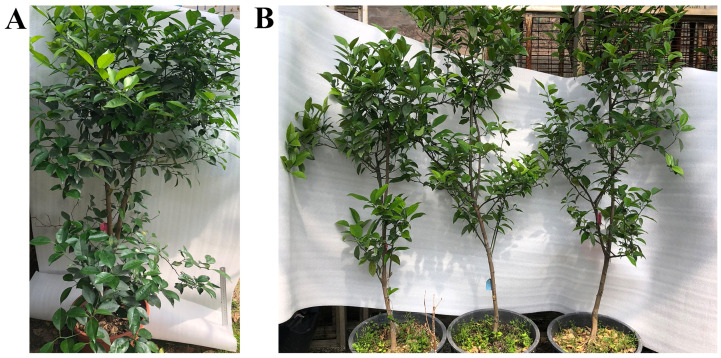
Phenotypes of transgenic plants after four years. (**A**). transgenic miR171b plants without HLB. (**B**). Transgenic miR171b plants with HLB. Non-transgenic plants with HLB died prior to four years after inoculation.

Three 171-5 grafted progenies and three non-transgenic controls were inoculated with HLB in November 2016, as described in the Materials and Methods. *C*Las was detected in leaves one month post-inoculation in the non-transgenic control plants, but was not detected in the transgenic plants until the 24th month (Figure 2). All three non-transgenic plants died less than two years after inoculation with HLB. In contrast, all three of the transgenic plants have survived to date, despite *C*Las being detectable since the 24th month (Figure 3). Two of the three transgenic plants began to fruit in the fifth year (2021); however, most of the fruit were abscised and only two were harvested in December.

### 2.3. Transcriptome Analysis via Illumina-Based RNA Sequencing

To understand the effect of overexpressing miR171b in relation to HLB resistance, both transgenic and non-transgenic plants were subjected to RNA-seq analysis. The mapped reads and uniquely mapped reads were more than 91.30% and 88.67%, respectively (Supplemental Table S3).

Differentially expressed genes (DEGs) among the treatments were identified using a *p* value ≤ 0.05 and |log_2_Ratio| ≥ 1 as the threshold. For the non-transgenic plants, 1770 and 1306 genes were up- and downregulated by overexpressing miRNA 171b, respectively, as shown in Supplemental Figure S1.

### 2.4. Gene Expression Validation Using qRT-PCR

The genes were randomly selected from the results of the RNA-Seq for the real-time fluorescence quantitative PCR detection, and the detection results were consistent with the RNA-Seq results, which indicates that the data of RNA-Seq were reliable (Supplemental Figure S2).

### 2.5. GO and KEGG Analysis

**Figure 4:**
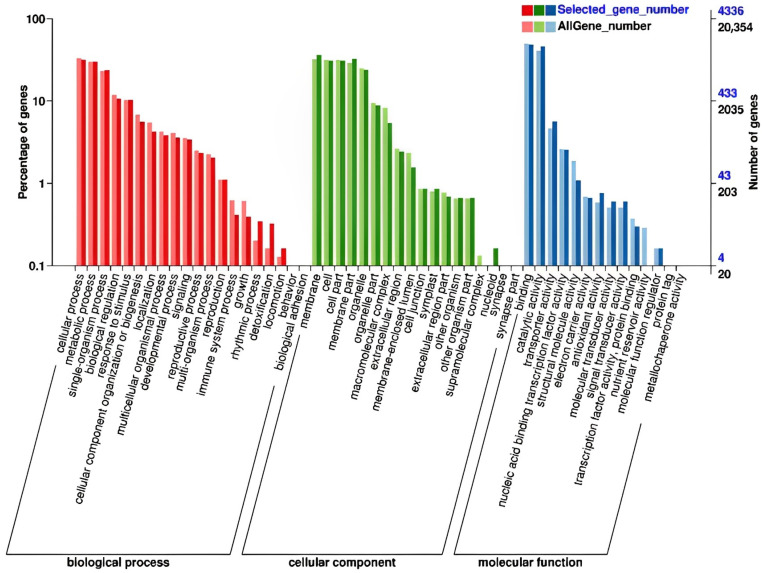
Summary of GO enrichment analysis. Biological processes, cellular components, and molecular functions are shown.

Based on gene ontology (GO) annotations, all of the DEGs were grouped into three major categories: biological processes, cellular components, and molecular functions. In the group of biological processes, most DEGs were involved in cellular processes, metabolic processes, single-organism processes, biological regulation, response to stimulus, cellular component organization or biogenesis, localization, multicellular organismal processes, developmental processes, signaling, reproductive processes, multi-organism processes, reproduction, immune system processes, growth, rhythmic processes, detoxification, and locomotion (Figure 4). In the functional group of cell components, most DEGs were associated with the cell, cell part, membrane, membrane part, organelle, organelle part, macromolecular complex, extracellular region, membrane-enclosed lumen, cell junction, symplast, extracellular region part, other organism, other organism part, supramolecular complex, and nucleoid. Lastly, in the group of molecular function, most DEGs were involved in binding, catalytic activity, transporter activity, nucleic acid binding transcription factor activity, structural molecule activity, electron carrier activity, antioxidant activity, molecular transducer activity, signal transducer activity, transcription factor activity, protein binding, nutrient reservoir activity, and molecular function regulation (Figure 4).

GO enrichment analysis of the DEGs revealed that GO terms were the most enriched in photosynthesis-, stress- and growth-related terms (Supplemental Table S4).

**Figure 5:**
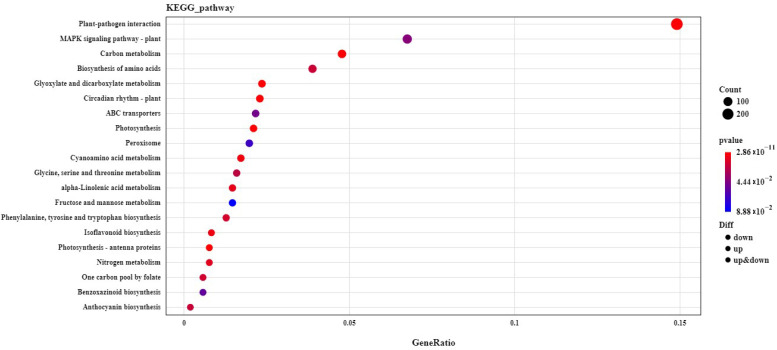
KEGG pathway enrichment analysis in the non-transgenic plants versus the transgenic plants.

Based on the comparison of the different groups, with the *p* value ≤ 0.05 as the standard, the enriched KEGG pathways are shown in Figure 5 and Supplemental Table S5. Most of the identified KEGG pathways were found to mainly be involved in photosynthesis, photosynthetic antenna proteins, plant–pathogen interactions, the MAPK signaling pathway, ABC transporters, and so on (Figure 5).

### 2.6. Analysis of the DEGs in the Photosynthesis Pathway

**Figure 6:**
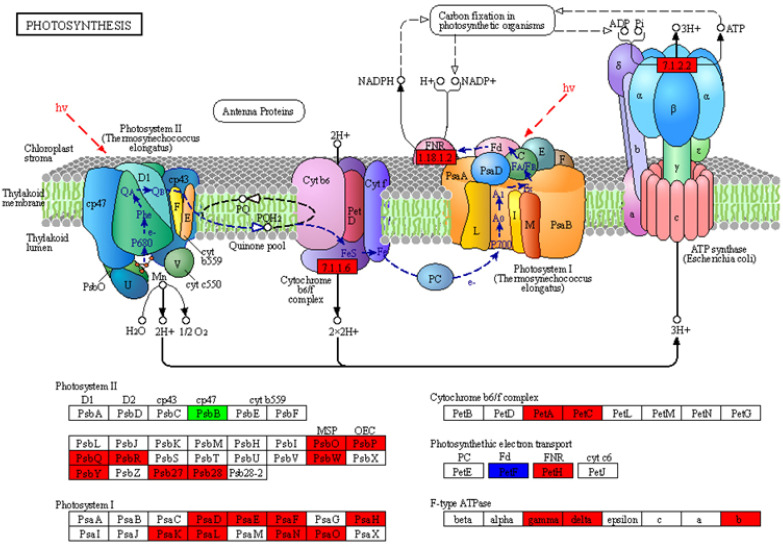
The KEGG map of the photosynthesis pathway in the non-transgenic plants versus the transgenic plants. The red means the DEGs were up-regulated, the green means the DEGs were down-regulated, and the blue means both up- and down- regulated.

**Figure 7:**
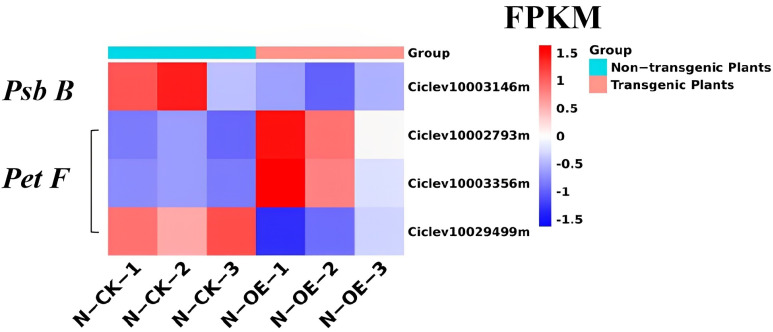
The FPKM of the DEGs in the photosynthesis pathway in the non-transgenic plants versus the transgenic plants. The N−CK−1−3 means the non-transgenic plants, the N−OE−1−3 means the transgenic plants. Other figures were the same.

The photosynthesis pathway was significantly changed in the non-transgenic plants compared to the transgenics; the DEGs related to photosystem I and photosystem II were all upregulated, except *PsbB* (Ciclev10003146m) and *PetF* (Ciclev10002793m, Ciclev10003356m, and Ciclev10029499m) (Figure 6). The *PsbB* associated with photosystem II was downregulated 1.24 times, and the FPKM of the non-transgenics was 10.05 and that of the transgenics was 3.66, with a slightly low expression level. The *PetF* genes were up- and down-regulated. The FPKM of the Ciclev10002793m ranged from 372.04 to 1530.69 and that of the Ciclev10003356m increased from 11.46 to 68.23, while only the FPKM of the Ciclev10029499m decreased from 343.86 to 125.27. The FPKM of some DEGs with respect to photosynthesis was higher in the transgenics than in the non-transgenics (Figure 7). The differences in the changes between the different samples seems to indicate that the transgenics were more able to store energy through photosynthesis in preparation for a response to HLB stress than non-transgenics.

### 2.7. Analysis of the DEGs in the Plant–Pathogen Interaction Pathway

**Figure 8:**
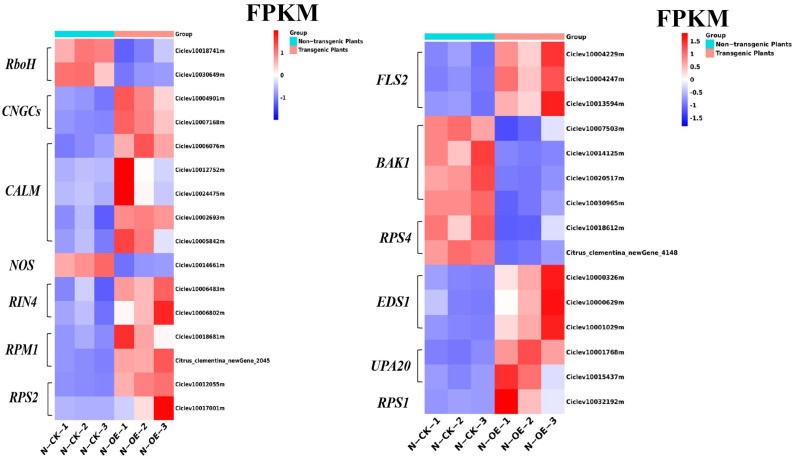
The FPKM of the DEGs in the plant–pathogen interaction pathway in the non-transgenic plants versus the transgenic plants.

The plant–pathogen interaction pathway exhibited a difference in the compared group. Comparing non-transgenic plants with transgenic plants, the *RboH* (Ciclev10018741m, Ciclev10030649m), which were associated with a hypersensitive response [38,39], were downregulated by 1.62 and 1.94 times, respectively, in the transgenic plants. The decrease in gene expression indicates that there was no HR response in transgenic plants in the absence of pathogen stress. In the transgenic plants, expression of the CNGC protein (Ciclev10004901m, Ciclev10007168m) in the Ca^2+^ pathway was up-regulated, with the FPKM increasing from 7.57 and 1.18 to 23.36 and 8.32, and the expressions of the *CALM* genes (Ciclev10006076m, Ciclev10012752m, Ciclev10024475m, Ciclev10002693m and Ciclev10005842m) were changed similarly, as shown in Figure 8, indicating that the *CALMs* were affected. The expression of the *NOS* gene (Ciclev10014661m) was downregulated, with FPKM falling from 5.01 to 1.70, although the level of gene expression was not significant.

Flagellin is a pathogen-associated molecular pattern that triggers the innate immune response in plants, in which *FLS2* (FLAGELLIN SENSITIVE2) is essential. In our results, for the transgenic plants, *FLS2* and *BAK1*, which are sensitive to pathogen infection, were activated, and the genes were up- and down-regulated, respectively. The *FLS2* genes (Ciclev10004229m, Ciclev10004247m and Ciclev10013594m) were upregulated, indicating that the immune response of the transgenic plant was activated.

The DEGs associated with disease resistance demonstrated significant changes in the transgenic plants. Among the genes, *RIN4* (Ciclev10006483m and Ciclev10006802m), which is an important susceptibility gene, was up-regulated 1.78 and 1.64 times, respectively. The FPKMs of the *RIN4* genes are shown in Figure 8, and the FPKM of Ciclev10006802m was much higher in the transgenic plants. At the same time, the *RPM1* gene, which interacts with the *RIN4* gene, was up-regulated, and both the *RPM1* and the *RIN4* genes were up-regulated simultaneously, suggesting that the *RIN4* network was activated in preparation for HLB stress. The *RPS2* disease-resistance genes (Ciclev10012055m and Ciclev10017001m) were also altered. The FPKM of *RPS2* is shown in Figure 8. The two genes of *RPS2* were both up-regulated. In addition to the *RIN4* gene, other genes involved in disease resistance, such as *RPS4* and *EDS1*, were also altered. The *EDS1* (Enhanced Disease Susceptibility 1) gene, as a core regulatory factor of plant immunity, regulates basic resistance by limiting pathogen invasion [40]. In our work, the *EDS1* genes included Ciclev10000326m, Ciclev10000629m, and Ciclev10001029m, which were up-regulated by 2.91, 1.86, and 2.94 times, respectively. Similarly, the FPKM of *EDS1* were identical to that of the *RIN4* gene, which was higher in the transgenic plants. *EDS1* was found to be an integral part of *RPS4* (Ciclev10018612m; Citrus_clementina_newGene_4148) signal transduction. The expression of *RPS4* was down-regulated, and the variation was consistent with *EDS1*. In addition, pathogen-related transcriptional factors such as *RPS1* (Ciclev10032192m) and *UPA20* (Ciclev10001768m, Ciclev10015437m) were all up-regulated, which was directly reflected in the FPKM values. In the plant–pathogen interaction pathway, the relevant genes were significantly altered in the majority of cases, suggesting that the plants overexpressing miR171b could enhance disease resistance for the plants at the base.

### 2.8. Analysis of the DEGs in the MAPK Pathway

The MAPK (mitogen-activated protein kinase) pathway plays an important role in transducing signals from upstream to downstream in the plant defense responses. A MAPK cascade usually contains a MAP kinase kinase kinase (MAPKKK), a MAP kinase kinase, and a MAPK [41,42]. From the results comparing non-transgenic plants with transgenic plants, it is evident that the MAPK pathway was significantly altered. The MEKK1 and MKK4/5 proteins of the MAPK cascade were both up-regulated in the transgenic plants. Compared with the non-transgenic plants, the *MEKK1* (Ciclev10002831m) increased by 1.32 times and the FPKM value increased from 2.71 to 6.78 in the transgenic plants. The expression of *MKK4/5* (Ciclev10016755m) was increased by 1.60 times, and the FPKM of Ciclev10016755m increased from 20.95 to 61.24. Other MAPK proteins, including MKK9, MAPKKK17/18, and MPK8, were all up-regulated.

**Figure 9:**
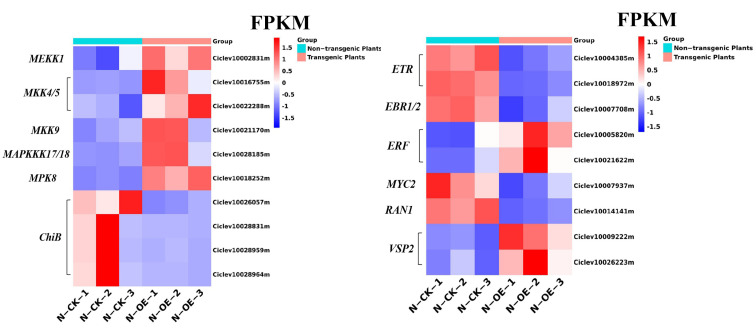
The FPKMs of the DEGs in the MAPK pathway in the non-transgenic plants versus the transgenic plants.

In plants, the MAPK cascades were also involved in a variety of physiological, developmental, and hormonal processes. As shown in Figure 9, in the ethylene pathway of the transgenic plants, the *ETR* (Ciclev10004385m, Ciclev10018972m), which encodes an ethylene receptor and acted upstream of *CTR1*, was down-regulated by 2.26 and 3.16 times, and the ethylene-related genes, *ERF* (Ciclev10005820m, Ciclev10021622m) and *EBF1/2* (Ciclev10007708m), were up-regulated and down-regulated, respectively. The DEGs in this pathway, such as *ChiB* (Ciclev10026057m, Ciclev10028964m, Ciclev10028959m and Ciclev10028831m) and *RAN1* (Ciclev10014141m), were all down-regulated. In the jasmonic acid pathway, the wounding-inducible expression of the *VSP2* (Ciclev10009222m, Ciclev10026223m) gene was up-regulated in the transgenic plants, and the stress-induced gene *MYC2* (Ciclev10007937m) was down-regulated. The FPKM of the *MYC2* gene ranged from 9 to 3, with low values. In the abscisic acid pathway, the related DEGs were all activated, which suggests that the hormones in the MAPK cascades were activated to enhance plant resistance.

**Figure 10:**
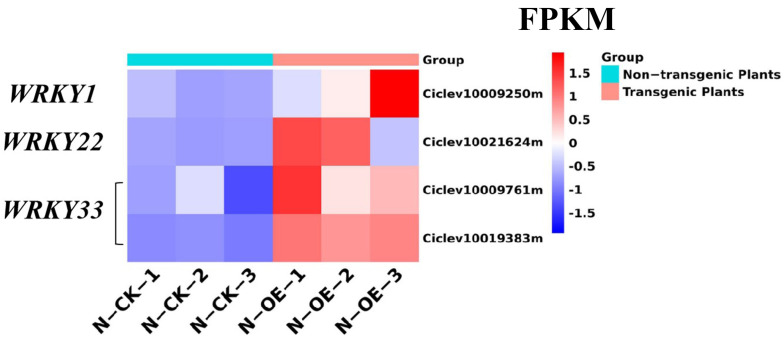
The FPKM of the WRKYs in the non-transgenic plants versus the transgenic plants.

In the MAPK pathway, *WRKY22* (Ciclev10021624m) and *WRKY33* (Ciclev10009761m, Ciclev10019383m), which belong to the WRKY superfamily of transcription factors involved in pathogen defense, were up-regulated in the transgenic plants (Figure 10). The WRKY proteins were involved in the biotic stress of plants, and several studies have shown that WRKY transcription factors play a regulatory role in the defense response of plants to pathogen infections [43]. The *WRKYs* in this pathway were all up-regulated. These results demonstrate that overexpression of the miR171b could activate the MAPK signal and enhance the resistance of transgenic plants through this pathway in the absence of pathogens.

### 2.9. Analysis of the DEGs in the Plant Hormone Signal Transduction Pathway

Several studies have shown that plant hormones are associated with citrus HLB. When the plants detect the pathogens, a network of plant hormones coordinate to carry out a defense response [44]. In this work, the plant hormone signal transduction pathway was not significantly altered. However, there were also 128 DEGs in this pathway, which may have enhanced our interpretation of these findings.

**Figure 11:**
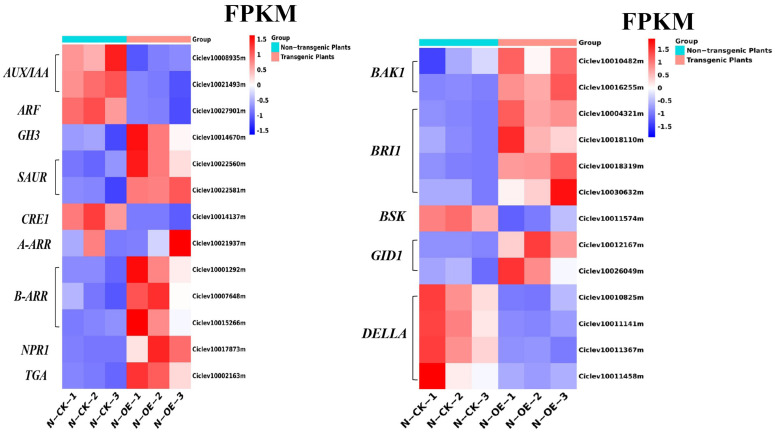
The FPKM of the DEGs in the plant hormone signal transduction pathway in the non-transgenic plants versus the transgenic plants.

Auxin plays an indispensable role in the growth and development of plants. The AUX/IAA protein is an auxin response inhibition factor that was activated in the samples, and the FPKM of this gene is shown in Figure 11. The downstream gene *ARF* (Ciclev10027901m) demonstrated the same trend as the *AUX/IAA* genes (Ciclev10008935m and Ciclev10021493m), which were all down-regulated in the transgenic plants, and the auxin-induced target genes *GH3* (Ciclev10014670m) and *SAUR* (Ciclev10022560m, Ciclev10022581m) were both up-regulated. Cytokinins play an important role in plant growth and development. In the transgenic plants, *CRE1* (Ciclev10014137m), which acts as a sensor for cytokinin, was down-regulated by 2.17 times. The cytokinin signal regulators *B-ARR* (Ciclev10001292m, Ciclev10007648m and Ciclev10015266m) and *A-ARR* (Ciclev10021937m) were both up-regulated at the same time. Brassinosteroids (BR) are a new class of plant steroid hormones, and increasing numbers of studies have shown that BR can improve resistance for plants. In our results, *BRI1* (brassinosteroid-insensitive 1) (Ciclev10004321m, Ciclev10018110m, Ciclev10018319m and Ciclev10030632m) and *BAK1* (*BRI1*-ASSOCIATED KINASE 1) (Ciclev10010482m and Ciclev10016255m) were changed at the same time, and the FPKM of these genes shows that the BR pathway was activated. However, the *BSK* (Ciclev10011574m) and BR signaling pathways downstream of *BRI1* was down-regulated by 1.24 times in the transgenic plants.

In this pathway, changes in the gibberellin signal pathway were noticeable. In the transgenic plants, the gibberellin receptor *GID1* (Ciclev10012167m; Ciclev10026049m) was up-regulated, the fold changes in *GID1* were 3.84 and 1.24 times, and the FPKMs increased from 9.89 and 5.40 to 144.09 and 12.23, respectively. The DELLA protein (Ciclev10010825m, Ciclev10011141m, Ciclev10011367m and Ciclev10011458m), which has a negative regulation effect on gibberellin [45], was obviously down-regulated; meanwhile, the FPKM indicated that the genes had been altered, which implies that gibberellin responds to HLB preparation in transgenic plants.

Salicylic acid (SA) is an important endogenous signal molecule in the activation of plant defense responses. In the SA signaling pathway, *NPR1* (Nonexpressor of pathogenesis-related genes 1) is a key regulator that plays a very important role [46]. In our results, *NPR1* (Ciclev10017873m) was up-regulated 1.70 times. The FPKM of the *NPR1* gene ranged from 23.52 to 72.77. The TGA protein (Ciclev10002163m), which interacted with the NPR1, was also up-regulated in the transgenic plants.

The pathways above were changed, which means that transgenic plants potentially provide a basis for growth through the photosynthesis-related pathways, and transform the plant–pathogen interaction, MAPK, plant hormones, and other pathways to enhance the tolerance of the transgenic plants and maintain the plant under HLB infection.

### 2.10. Predicted Targets of miRNA171b

The candidate genes were predicted to be the targets of miR171b (Table 1). Using rigorous screening parameters, the four candidate target genes (Cs5g08980.1, Orange1.1t00199.1, Orange1.1t00200.1, and Orange1.1t00200.2) were identified. They were all found to belong to *SCL6* gene homologs.

### 2.11. Expression of the Genes Targeted by miR171b

**Figure 12:**
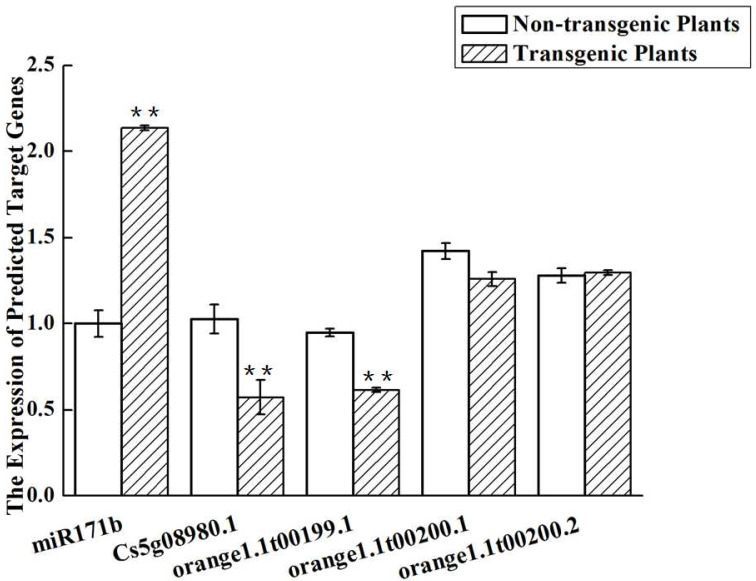
The expression level of the predicted target genes for miR71b. Experiments were repeated twice with similar results. ** indicates *p* values of <0.01 compared with the non-transgenic plants.

qPCR was used to compare the expression of the assumed target genes of miR171b in the transgenic and non-transgenic plants. Two of the four predicted targets were significantly downregulated (Figure 12). More specifically, orange1.1t00199.1 expression was reduced by 1.6-fold and Cs5g08980.1 expression was reduced by approximately 2-fold in the transgenic plants. The expressions of the other two predicted target genes did not differ significantly. The Cs5g08980.1 and the orange1.1t00199.1 genes were all the target genes of miR171b.

## 3. Discussion

miRNAs play significant roles not only in plant growth and development [19,25], but also in plant defense against abiotic and biotic stresses [47]. It is therefore not surprising to find that significant changes occurred in the expression of some miRNAs in citrus following infection with *C*Las, as shown in our previous study [37]. Although not all of these HLB-modulated miRNAs are necessarily involved in the responses of citrus to HLB, one class, miR171, caught our eye, for its homologs are involved in plant–microbe interactions in other plants. For example, miR171h in *Medicago truncatula* controls fungal colonization by downregulating the GRAS transcription factor *NSP2*, a member of the super *SCL* gene family [48]. In wheat (*Triticum aestivum* L.), miR171 demonstrated differential expression patterns in response to powdery mildew fungal infection [49]. In soybean, miR171o and miR171q affect the expression of *SCL6* and *NSP2* during the rhizobi–soybean nodulation process [50]. Recently, miR171 was found to be involved in *Citrus psorosis virus* infection in citrus [36]. Therefore, we investigated the possible role of miR171b in the response of citrus to HLB. The precursor gene of miR171b was cloned from orange and transgenic plants overexpressing the gene were generated. The clonal progenies of the transgenic plants were challenged with *C*Las bacteria in 2016. It was a surprise to us that no detectable *C*Las was found by PCR until the 24th month of infection, and that the plants have survived to date. Contrastingly, all non-transgenic plants collapsed from *C*Las within two years. These results clearly indicate that miR171b positively regulated the resistance to HLB of citrus.

The results from RNA-seq sequencing, by comparing the enrichment of DEG pathways in the transgenic plants versus the non-transgenics, indicate that the pathways are similar in the two biological groups, such as the photosynthesis and photosynthetic antenna protein pathways. Among the photosynthesis pathway, the DEGs were most involved in photosystem I and photosystem II. The DEGs in the photosynthetic antenna pathway were found to be involved in chlorophyll a-b binding protein. A change in photosynthetic machinery is common amongst the various pathogenic responses [51]. The inhibition of photosynthesis has already been found during the pathogenesis of *Botrytis cinerea* in plants such as Arabidopsis, tomato, and lettuce [52,53]. Thus, DEGs in the photosynthetic pathway were active in both transgenic plants and control plants for the same purpose of providing energy for plant survival, but the changes in the genes related to chlorophyll metabolism seem to explain why the leaves mottled after being infected with HLB bacteria.

Pathways related to disease resistance, such as plant–pathogen interactions, the MAPK signaling pathway, and ABC transporters, were all activated in transgenic plants. In the plant–pathogen interaction pathway, 234 DEGs were up/down-regulated, including the disease-related genes, such as *RIN4*, *EDS1*, *WRKYs*, and others. A total of 106 DEGs were involved in the MAPK signaling pathway, and MAPK has been shown to be involved in plant disease resistance. Li et al. found that flg22-MEKK1-MKK4/5-MPK3/6-WRKY22/29 could promote the expression of resistance genes by using the transient expression system of *Arabidopsis* protoplasts [54]. It was also found that flg22-induced *MPK4* activity was significantly reduced in *Arabidopsis mekk1* mutants, while the activity of *MPK3/6* activity was not affected. These studies suggest that the *MEKK1-MKK4/5-MPK3/6-WRKY22/29/33* cascade pathway is involved in the resistance response to *Arabidopsis thaliana*. In this work, the related genes *MEKK1-MKK4/5-WRKY22* were up-regulated, but *MPK3/6* was unchanged. The above may be one of the reasons for the promotion of resistance to HLB in the transgenic plants.

Smirnova et al. found that genes were expressed differently in pathogen invasion [55]. Furthermore, gene expression patterns changed the metabolism pathways and pathogen defense systems [56]. Although there was no significant difference in plant hormone signal transduction, a total of 128 DEGs were associated with this pathway. The *DELLA* gene, a key gene for inhibiting GA response, was altered in the GA pathway. The *GID1*, *GID2,* and *DELLA* genes were all altered in comparison with the non-transgenic plants versus the transgenic plants, which seems to indicate that the GA pathway was prepared for stress in the transgenic plants. Ma et al. found that *SCL27*, which was the target gene of miR171, interacted with DELLA, suggesting that miR171-*SCL* plays an important role in GA signaling [57]. Ma et al. discovered that the addition of gibberellin alleviated the stress of HLB by upregulating the genes encoding the ROS-producing NADPH oxidase and down-regulated antioxidant enzyme genes [58]. In this study, the *DELLA* gene was altered in transgenic plants; it may be related to the regulation of the target gene by miR171b, and also indicates that miR171b may be involved in the regulation of the GA signaling pathway.

Salicylic acid (SA) is an important phytohormone that regulates plant growth, environmental stress, and pathogen stress. Within a few years after SA was first discovered to play a role in heat production, SA was also found to be used as a signaling molecule during pathogen infection [59,60]. In apple (*Malus domestica*), exogenous SA could enhance the resistance to Glomerella leaf spot (GLS) [61]. The *NPR1* gene is a key regulator in the SA signal transduction pathway. The overexpression of *AtNPR1* resulted in citrus trees with normal phenotypes exhibiting enhanced resistance to HLB [62]. According to our results, the *NPR1* gene and *TGA* gene expression were both upregulated in the SA pathway, which indicates that the salicylic acid pathway was activated for the overexpression of miR171b.

To our knowledge, miRNAs function through silencing complementary mRNA to regulate the expression of target genes. The miR171 family primarily targets the GRAS transcription factor gene family and regulates genes at the post-transcriptional level [63,64]. *SCL* (Scarecrow-like, including *SCL*, *NSP2*, *NORK*, etc.) is a core member of the GRAS family. In the model plant *Arabidopsis thaliana*, miR171 was found to slice the *AtSCL6*, *AtSCL22*, and *AtSCL27* genes [28,63]. In recent years, *GRAS* genes have also been connected with plant disease resistance and responses to abiotic stress [28,40,65,66]. For example, *GRAS* genes play a role in pathogen resistance in tomato (*Solanum lycopersicum*) [63]. The expression of *S1GRAS4* and *S1GRAS6* was induced by the fungal elicitor EIX in tomato [63]. Similarly, the application of H_2_O_2_ to tobacco induced the expression of *GRAS* genes, demonstrating their involvement in plant protection against pathogens [67,68]. In chickpeas infected by *Pseudomonas putida*, miR171 down-regulated the target gene *NPS2*, which affected other rhizosphere-beneficial bacteria and promoted the growth of chickpea roots under drought stress [29]. In this study, bioinformatics was used to identify the target genes of miR171b in citrus. We observed a decrease in the expression of the target genes Cs5g08980.1 and orange1.1t00199.1, likely due to these genes being the target genes of miR171b, but verification of this is the next step. These results suggest that miR171b may increase resistance to citrus HLB through the target genes as well. However, the mechanism of how miR171b interacts with the *SCL6* gene to improve resistance to citrus HLB remains unknown, and will be the target of future research.

In the current study, we provide evidence that miR171b plays a positive regulatory role in increasing citrus resistance to HLB through the downregulation of its target genes and multiple metabolic and biological pathways. This study is the first to report the regulation of HLB resistance at the level of microRNAs, and may represent a new molecular mechanism for the regulation of HLB stress adaption.

## 4. Materials and Methods

### 4.1. Cloning of pre-ctr-miR171b

Genomic DNA was extracted from leaves of trifoliate orange (*Citrus trifoliata*) using a DNA extraction kit (Dong sheng Biotechnology, Guangzhou, China). PCR primers are listed in Supplemental Table S1. The ctr-miR171b precursor (pre-ctr-miR171b) was PCR amplified using the 171f/171r primer pair and KOD FX DNA polymerase (Toyobo, Shanghai, China). The precursor sequence was downloaded from miRbase (http://www.mirbase.org/cgi-bin/browse.pl) (accessed on 3 August 2015). The amplified product was purified, ligated into the pEASY-T1 vector (TransGen Biotechnology Co., Ltd., Beijing, China), and cloned into *Escherichia coli* strain Trans1-T1. Positive clones were used to extract plasmids using a Vazyme plasmid purification kit (Vazyme Biotech Co., Ltd., Nanjing, China), and the correct inserts were verified by sequencing several plasmids with M13F/R primers.

### 4.2. Construction of Expression Vectors

A plasmid containing the correct ctr-miR171b precursor insert was digested with SacI and BamHI to release the insert. The digested materials were separated via electrophoresis, and the insert was recovered from the gel using a TaKaRa MiniBEST Agarose Gel DNA Extraction Kit (Takara, Dalian, China). The precursor was then ligated using T4 ligase into the linearized vector (pFGC5941) and prepared via digestion with SacI and BamHI. The ligated product was transformed into E. coli competent cells purchased from TransGen (Beijing, China) using the freeze–thaw method. Positive clones were extracted using a Vazyme plasmid purification kit (Nanjing, China) and verified using PCR.

### 4.3. Genetic Transformation and Identification of Transgenic Plants

Citrus transformation was performed according to the method described by Cheng et al. [69] using Citrus reticulata Blanco “tangerine” epicotyls as explants. *Agrobacterium* infection solution was prepared and the OD_600_ was adjusted to 0.5. The epicotyls of citrus were cultured in the dark, and the epicotyls were diagonally cut into sections of 1~2 cm, which were placed in *Agrobacterium* infection solution (containing 100 µM Acetosyringone) and infected for 30 min. The explants were removed and incubated in MS medium at 25 °C for 3 days. The explants were transferred to screening medium containing 10 mg/L Basta and cultured at 28 °C for 30 to 45 days. When the resistant regeneration buds had grown to 2 cm, the whole regeneration buds, together with the epicotyls, were grafted onto the stocks. Genomic DNA was isolated from fully expanded leaves of the regenerated plants and examined for the presence of the transgene via PCR using the pFGC5941 vector specific primer pair P1/P2 flanking the expression cascade. Further verification was carried out using PCR with the upstream *35S*-F primer and the downstream 171r primer. The PCR-positive transgenic plants were propagated by grafting onto red tangerine rootstocks. The graft-propagated transgenic plants were re-examined using *35S*-F/*35S*-R and Bar-F/Bar-R primers. Non-transgenic plant buds were simultaneously graft-propagated and used as controls.

### 4.4. Analysis of miR171b Expression Levels

Small RNA (sRNA) was isolated from the leaves of transgenic plants using the RNAiso for Small RNA kit (Takara, Dalian, China) according to the manufacturer’s instructions, and complementary DNA (cDNA) synthesis was performed using the miRcute Plus miRNA First-Strand cDNA kit (TianGen Biotechnology Co., Ltd., Beijing, China). miR171b was quantified using quantitative real-time PCR (qPCR) with the SYBR Green Premix for miRNA (TianGen Biotechnology Co., Ltd., Beijing, China) on the QuantStudio5 Real-Time PCR System (Thermo Fisher Scientific).

The transcription levels of miR171b were expressed as relative ratios between qPCR values of miR171b and the reference small RNA U6 using the 2^−ΔΔCT^ method [70], and P-miR171b and P-U6 were used as primers. The thermo cycler was programmed as follows: pre-incubation at 95 °C for 30 s followed by 45 cycles of denaturation at 95 °C for 5 s and annealing at 60 °C for 20 s. Three technical replicates were used in this study. The differences in expression between transgenic and non-transgenic plants were considered significant when *p* < 0.05 and very significant when *p* < 0.01.

### 4.5. Determining the Ct Values of HLB Bacteria

Transgenic plants and non-transgenic controls were infected with *C*Las by grafting with HLB-infected buds. Three biological replicates were used. qPCR with the TaqMan probe method was used to quantify HLB DNA in the transgenic plants at an interval of 30 d in the first year, 90 d in the following three years, and 180 d in the remaining years. The positive and negative controls (HLB diseased and healthy trees, respectively) were confirmed based on the symptoms and by using PCR with HLB-specific primers. DNA was extracted from the leaves of the samples using a plant genomic DNA Rapid Extraction Kit (Dongsheng, Guangzhou, China). The DNA samples were diluted to uniform concentrations and used as the template to titer *C*Las bacteria in the samples. A Ct value of >36 was used as the cutoff for HLB-negative samples because noisy signals from non-specific amplifications occurred frequently above this value [71,72].

### 4.6. Plant Materials, RNA Isolation, and Quantification

Five-year-old transgenic trees expressing miRNA 171b precursor and non-transgenic trees were used as the material for RNA-seq, with three biological replicates for each. All trees were planted in the greenhouse at the Institute of Fruit Research, Guangdong Academy of Agricultural Sciences. Mature leaves from the plants of the two compared groups with the three replicates were used to extract total RNA by using an RNA extraction kit (RP3202, polysaccharide and polyphenol total RNA isolation kit, BioTeke, Beijing, China). RNA quality control and inspection used agarose gel (1.5%) electrophoresis and spectrophotometer measurements (NanoDrop 2000, Thermo Fisher Scientific, Lafayette, CO, USA). Three biological samples were used. Then, the libraries were constructed using the VAHTS Universal V6 RNA-seq Library Prep Kit according to the manufacturer’s instructions. The transcriptome sequencing and analysis were conducted by Biomarker Technologies Co. (Beijing, China).

### 4.7. RNA-Seq Experiment

Total RNA was enriched for mRNA with Oligo(dT) beads, while the mRNA was enriched by removing rRNA using the Ribo-Zero^TM^ Magnetic Kit (Epicentre, Madison, WI, USA). Then, the enriched mRNA was fragmented into short fragments using fragmentation buffer and reverse transcribed into cDNA with random primers. Second-strand cDNA was synthesized using DNA polymerase I, RNase H, dNTP, and buffer. Then, the cDNA fragments were purified with the QiaQuick PCR extraction kit (Qiagen, Venlo, The Netherlands), end repaired, poly(A) added, and ligated to Illumina sequencing adapters. The ligation products were selected using agarose gel electrophoresis, PCR amplified, and sequenced using Illumina HiSeq2500 by Biomarker Technologies Co. (Beijing, China) and 150 bp paired-end reads were generated. The raw reads for each sample were generated. Raw reads of fastq format were firstly processed using fastp, and the low-quality reads were removed to obtain the clean reads. Then, the clean reads for each sample were retained for subsequent analyses. The clean reads were mapped to the reference genome using HISAT2. Fragments Per Kilobase per Million (FPKM) of each gene was calculated and the read counts of each gene were obtained by HTSeq-count.

Differential expression analysis was performed using the DESeq2. Hierarchical cluster analysis of DEGs was performed using R (v 3.2.0) to demonstrate the expression pattern of genes in different groups and samples. Based on the hypergeometric distribution, GO and KEGG pathway enrichment analysis of DEGs was performed to screen the significantly enriched terms using R (v 3.2.0). R (v 3.2.0) was used to draw the column diagram and bubble diagram of the significant enrichment term.

### 4.8. Validation of DEGs in a qRT-PCR Assay

The relative expression levels of genes in citrus were detected via qRT-PCR using a previously described method. The thermo cycler was programmed as follows: pre-incubation at 95 °C for 10 min followed by 40 cycles of denaturation at 95 °C for 5 s, 60 °C for 20 s, 95 °C for 15 s and 60 °C for 1 min. All of the qRT-PCR experiments were performed with three technical replicates each. PCR primers and the genes info are listed in Supplemental Table S2.

### 4.9. Bioinformatics Analysis of miR171b Target Genes

The website https://www.zhaolab.org/psRNATarget/home, accessed on 15 October 2020 was used for the analysis of the miRNA target genes. Targets were identified by restricting mismatches to <3 nt and gaps to <1 nt. More rigorous screening parameters were used to narrow down the target genes: (1) less than or equal to three mismatches between mature miRNA and its target sites; (2) less than or equal to one mismatch on the first 1–9 nt between the target and the mature miRNA; (3) no mismatches at the 10–11 nt between the target and the mature miRNA; and (4) no mismatches at the base-pairing region between the target and the miRNA.

### 4.10. Data Analysis

Statistical analysis was performed using SPSS (version 17.0; https://www.ibm.com/analytics/data-science/predictive-analytics/spss-statistical-software). (accessed on 9 August 2020). Between-group comparisons were made using one-way analysis of variance (ANOVA) followed by a Tukey’s multiple range test. The mean ± SD of the three replicates were calculated. *p* < 0.05 and *p* < 0.01 were considered statistically significant or very statistically significant, respectively.

## 5. Conclusions

In general, this study demonstrates that overexpressing miR171b enhanced resistance to HLB through relevant pathways and related target genes. Moreover, this study provides novel insights into the molecular breeding of citrus against HLB.

## Figures and Tables

**Table 1:** The predicted target genes of miR171b.

Gene ID	Gene Info
orange1.1t00200.1	Scarecrow transcription factor family protein; Scarecrow-like protein 6; DELLA protein RGA
orange1.1t00200.2	GRAS family transcription factor containing protein; Scarecrow-like protein 6; DELLA protein RGA
Cs5g08980.1	GRAS family transcription factor containing protein; Scarecrow-like protein 6
orange1.1t00199.1	GRAS family transcription factor containing protein; Scarecrow-like protein 6

## Data Availability

RNA-seq data have been submitted to the NCBI Sequence Read Archive (SRA). https://www.ncbi.nlm.nih.gov/sra/PRJNA906724 (accessed on 30 November 2022).

## Supplementary Materials

The following supporting information can be downloaded at: https://www.mdpi.com/article/10.3390/ijms24065737/s1.
