# Supplementary material for: MicroRNA miR171b Positively Regulates Resistance to Huanglongbing of Citrus
Source: Int J Mol Sci. 2023 Mar 17;24(6):5737. doi: 10.3390/ijms24065737 (PMC10053592; doi:10.3390/ijms24065737)
Supplement: Supplementary file 1 [file ijms-24-05737-s001.zip › Figrues and Tables Captions.pdf]

Figure S1: Visualized volcano map of DEGs in the non-transgenic plants versus the transgenic plants.

Figure S2: Gene expression validation using qRT-PCR. (**A**,**B**): The expression of selected genes; (**C**): Results of RNA-seq data.

Table S1: The primers of this work.

Table S2: The information of the selected genes.

Table S3: Summary of the transcriptome assembly of total reads.

Table S4: Enriched gene ontology terms of differentially expressed genes in non-transgenic plants versus transgenic plants.

Table S5: Summary of KEGG pathway enrichment.
