# Supplementary material for: MicroRNA miR171b Positively Regulates Resistance to Huanglongbing of Citrus
Source: Int J Mol Sci. 2023 Mar 17;24(6):5737. doi: 10.3390/ijms24065737 (PMC10053592; doi:10.3390/ijms24065737)
Supplement: Supplementary file 1 [file ijms-24-05737-s001.zip › Supplemental Table S1.pdf]

Tables

Supplemental Table S1. The primers of this work.

Primer	sequence
171f	TTggcgcgccAAtctagaATCAATATGACAGG AAT
171r	CTAGactagtTGCTTAAAAGAAAGC
M13F	TGTAAAACGACGGCCAGT
M13R	CAGGAAACAGCTATGACC
P1	AGCAAGTGGATTGATGTGAC
P2	GGTAAGGATCTGAGCTACAC
35S-F	TCATAAACCAAGGCAAGTAATAGAG
35S-R	GATAGTGGGATTGTGCGTCAT
BAR-F	TGCACCATCGTCAACCACTACATC
BAR-R	GCTGCCAGAAACCCACGTCAT
P-miR171b	TGATTGAGCCGTGCCAATATC
P-U6	ACAGAGAAGATTAGCATGGCC
HLB-Probe	AGACGGGTGAGTAACGCG
HLB-F	TCGAGCGCGTATGCAATAC
HLB-R	GCGTTATCCCGTAGAAAAAGG
Cs5g08980.1-F	TCGGTGCTCAGTGGGCTTCATTTAT
Cs5g08980.1-R	AATGGGCAGTGTGTAAGAGGTTTGA
Orange1.1t00199.1-F	CTTCGTCCACACATGATGAGTTTGA
Orange1.1t00199.1-R	TCTTGGGTTGAAGTTGCTTTACG
Orange1.1t00200.1-F	AAAGTGAGGCAACTGCTGTGAAT
Orange1.1t00200.1-R	GGAGTATGACTGAAGTGCGTGAA
Orange1.1t00200.2-F	TTTGCTTCTCCGTCCACACATGATG
Orange1.1t00200.2-R	GCAGTTGCCTCACTTTCTAATCCCC
