# Supplementary material for: MicroRNA miR171b Positively Regulates Resistance to Huanglongbing of Citrus
Source: Int J Mol Sci. 2023 Mar 17;24(6):5737. doi: 10.3390/ijms24065737 (PMC10053592; doi:10.3390/ijms24065737)
Supplement: Supplementary file 1 [file ijms-24-05737-s001.zip › Supplemental Table S2.pdf]

Tables

Supplemental Table S2. The information of the selected genes.

Gene ID	Primer	Gene Info	KEGG_pathway_annotation
Ciclev10017873m	ACAGTGGAGATGGGTCAGCGTTAT	NPR1/NIM1 like defence protein C terminal	Plant hormone signal transduction (ko04075)
	CTTTGTCCCTTGTAAATGCCTTCTG		
Ciclev10000733m	GAACAAGAAACATTATCCTCCATC	calmodulin-binding protein	--
	ATACAACTTCCCATCATTTACGAC		
Ciclev10002163m	TTTCGAAGCCCTAATTACAGACCTC	DnaJ domain signature	Plant hormone signal transduction (ko04075)
	GCTCAAGAGTTTCGTATGGAGATTT		
Ciclev10029691m	AAGCTCACCAAGAAGCAAAACCG	hypothetical protein	--
	TTCCCCAAAATCAGACCCAAAAT		
Ciclev10015194m	TGCCCGTATTTGAACCAACTGCT	Transferase family	--
	TACCAAACCAGAACCATCACACATT		
Ciclev10021802m	CTCAATACTGCCATCCGCTGCTT	RING-H2 finger protein	--
	CCTCTGCTCCTGTTAATTTGATCCC		
Ciclev10005194m	GCGTTATCTGCCACTACTATCCG	2OG-Fe (II) oxygenase superfamily	Benzoxazinoid biosynthesis (ko00402)
	TATTTACCACCAAGCCTCCTGAC		
Ciclev10014968m	AACTCAGCCCTTTTCTTGCTTTCTC	Mate efflux family protein	--
	GCCTCCAATCATCCCAGACCATA		
Ciclev10002075m	ACCCATTTGGAGCCTCTACTACTGA	Protein tyrosine and serine/threonine kinase	--
	AATTTGGCCGCATATTCTTCCTG		
Ciclev10025151m	TCTCCAACTACCCAATACCAAACAT	Leucine rich repeat	--

	CATCTCACCAACCGAAGAAGGAATA		
Ciclev10013959m	TCTCCATTGGTATGAATATCCTTTA<br>TGTATTGAGGAATGTAAGCGAAG	Disease resistance protein (NBS-LRR-CLASS) family protein	Plant-pathogen interaction (ko04626)
Ciclev10020440m	ATCAGTTCATTCGGCAAACCAGC<br>GACCCGTCTTCACAGCCAATCTC	B-box zinc finger protein	Circadian rhythm - plant (ko04712)
Ciclev10019516m	GACTTGGAAACATCACGGGCTAA<br>TTAGCAGAAGGGTCGACAAAGTACA	U3 small nucleolar RNA-associated protein 18 homolog	Ribosome biogenesis in eukaryotes (ko03008)
Ciclev10002732m	CCTAATTCAGAAGGCGGATACTAACA<br>ATCCATTGCCATCCCTATCAAACAT	EF-hand calcium-binding protein	Plant-pathogen interaction (ko04626)
Ciclev10033073m	AAAGGAGACGGGAGCATAACGGAGA<br>TATCCGTCAACTTATGCCGTGCCTC	NPR1 interacting protein	--
Ciclev10017843m	TGGCTACCTTGGGACCTTATCTT<br>ATGCTTCCTCTACCCTCAACCTG	THO2 Protein	RNA transport (ko03013); Spliceosome (ko03040)
Ciclev10026870m	GGCTAAGGTTAAGGTTGCCACTC<br>CTTCTCCGTCACAGTCATCGTCT	Phytosulfokine precursor protein	--
Ciclev10004122m	AGAAGTATTGAATGGGCAGAGTC<br>CAAGCAAGTATTTGAAGTAGGGA	BP28CT (NUC211) domain	Ribosome biogenesis in eukaryotes (ko03008)
Ciclev10009222m	CAAGGTGCGTCGAGTTCGTTCAG<br>TAGGGCAAGTTGGAAAGAAGGGT	HAD superfamily, subfamily IIIB (Acid phosphatase)	MAPK signaling pathway - plant (ko04016)
Ciclev10019263m	GACAAGGTCACCAAGGGTCAAGT<br>TCCCGTAATCCCAATAACAGTCC	Lecithin:cholesterol acyltransferase	Glycerolipid metabolism (ko00561)
