# Supplementary material for: MicroRNA miR171b Positively Regulates Resistance to Huanglongbing of Citrus
Source: Int J Mol Sci. 2023 Mar 17;24(6):5737. doi: 10.3390/ijms24065737 (PMC10053592; doi:10.3390/ijms24065737)
Supplement: Supplementary file 1 [file ijms-24-05737-s001.zip › Supplemental Table S3.pdf]

**Tables**

**Supplemental Table S3. Summary of the transcriptome assembly of total reads.**

| Categories/ Raw data | Total reads | mapped Reads | Uniq Map |
|---|---|---|---|
| N-CK-1 | 42827360 | 40029162 (93.47%) | 38825690 (90.66%) |
| N-CK-2 | 55617630 | 52090629 (93.66%) | 50713507 (91.18%) |
| N-CK-3 | 43253056 | 40548746 (93.75%) | 39534589 (91.40%) |
| N-OE-1 | 51805682 | 48561338 (93.74%) | 46943514 (90.61%) |
| N-OE-2 | 38591158 | 35948496 (93.15%) | 34917060 (90.48%) |
| N-OE-3 | 53022910 | 48411252 (91.30%) | 47015099 (88.67%) |
