# Supplementary material for: MicroRNA miR171b Positively Regulates Resistance to Huanglongbing of Citrus
Source: Int J Mol Sci. 2023 Mar 17;24(6):5737. doi: 10.3390/ijms24065737 (PMC10053592; doi:10.3390/ijms24065737)
Supplement: Supplementary file 1 [file ijms-24-05737-s001.zip › Supplemental Table S4.pdf]

Tables

Supplemental Table S4. Enriched Gene ontology term of differentially expressed genes at non-transgenic plants versus transgenic plants.

GO-ID	p-value	q-value	Description
16168	3.05E-13	3.47E-10	chlorophyll binding
5524	4.91E-13	3.47E-10	ATP binding
8194	3.81E-09	1.35E-06	UDP-glycosyltransferase activity
9523	3.83E-09	1.35E-06	photosystem II
9522	4.18E-07	1.18E-04	photosystem I
9538	1.09E-06	2.57E-04	photosystem I reaction center
15979	1.36E-06	2.73E-04	photosynthesis
9611	2.65E-06	4.16E-04	response to wounding
8152	2.65E-06	4.16E-04	metabolic process
10051	3.90E-06	0.000913	xylem and phloem pattern formation
45927	2.39E-05	0.002545	positive regulation of growth
34097	0.00049	0.036341	response to cytokine
