# Supplementary material for: MicroRNA miR171b Positively Regulates Resistance to Huanglongbing of Citrus
Source: Int J Mol Sci. 2023 Mar 17;24(6):5737. doi: 10.3390/ijms24065737 (PMC10053592; doi:10.3390/ijms24065737)
Supplement: Supplementary file 1 [file ijms-24-05737-s001.zip › Supplemental Table S5.pdf]

Tables

Supplemental Table S5. Summary of KEGG pathway enrichment.

Gene ID	Description	P-value	q-value	Gene_number
ko00195	Photosynthesis	2.86E-11	3.37E-09	33
ko00630	Glyoxylate and dicarboxylate metabolism	1.07E-06	6.28E-05	37
ko00196	Photosynthesis - antenna proteins	2.10E-06	8.24E-05	12
ko04712	Circadian rhythm - plant	3.15E-06	9.29E-05	36
ko04626	Plant-pathogen interaction	0.000706	0.016646	234
ko01200	Carbon metabolism	0.001062	0.020862	75
ko00460	Cyanoamino acid metabolism	0.004813	0.081062	27
ko00943	Isoflavonoid biosynthesis	0.005968	0.08795	13
ko00592	alpha-Linolenic acid metabolism	0.008739	0.114478	23
ko00910	Nitrogen metabolism	0.009954	0.117347	12
ko00400	Phenylalanine, tyrosine and tryptophan biosynthesis	0.014899	0.157342	20
ko00670	One carbon pool by folate	0.016084	0.157342	9
ko01230	Biosynthesis of amino acids	0.018337	0.157342	61
ko00942	Anthocyanin biosynthesis	0.018684	0.157342	3
ko00260	Glycine, serine and threonine metabolism	0.023029	0.181004	25
ko04016	MAPK signaling pathway - plant	0.041903	0.308756	106
ko02010	ABC transporters	0.048938	0.339386	34
ko00402	Benzoxazinoid biosynthesis	0.054357	0.35602	9
ko04146	Peroxisome	0.068405	0.424453	31
ko00051	Fructose and mannose metabolism	0.088827	0.523614	23
