# Supplementary figures and images for: MicroRNA miR171b Positively Regulates Resistance to Huanglongbing of Citrus
Source: Int J Mol Sci. 2023 Mar 17;24(6):5737. doi: 10.3390/ijms24065737 (PMC10053592; doi:10.3390/ijms24065737)

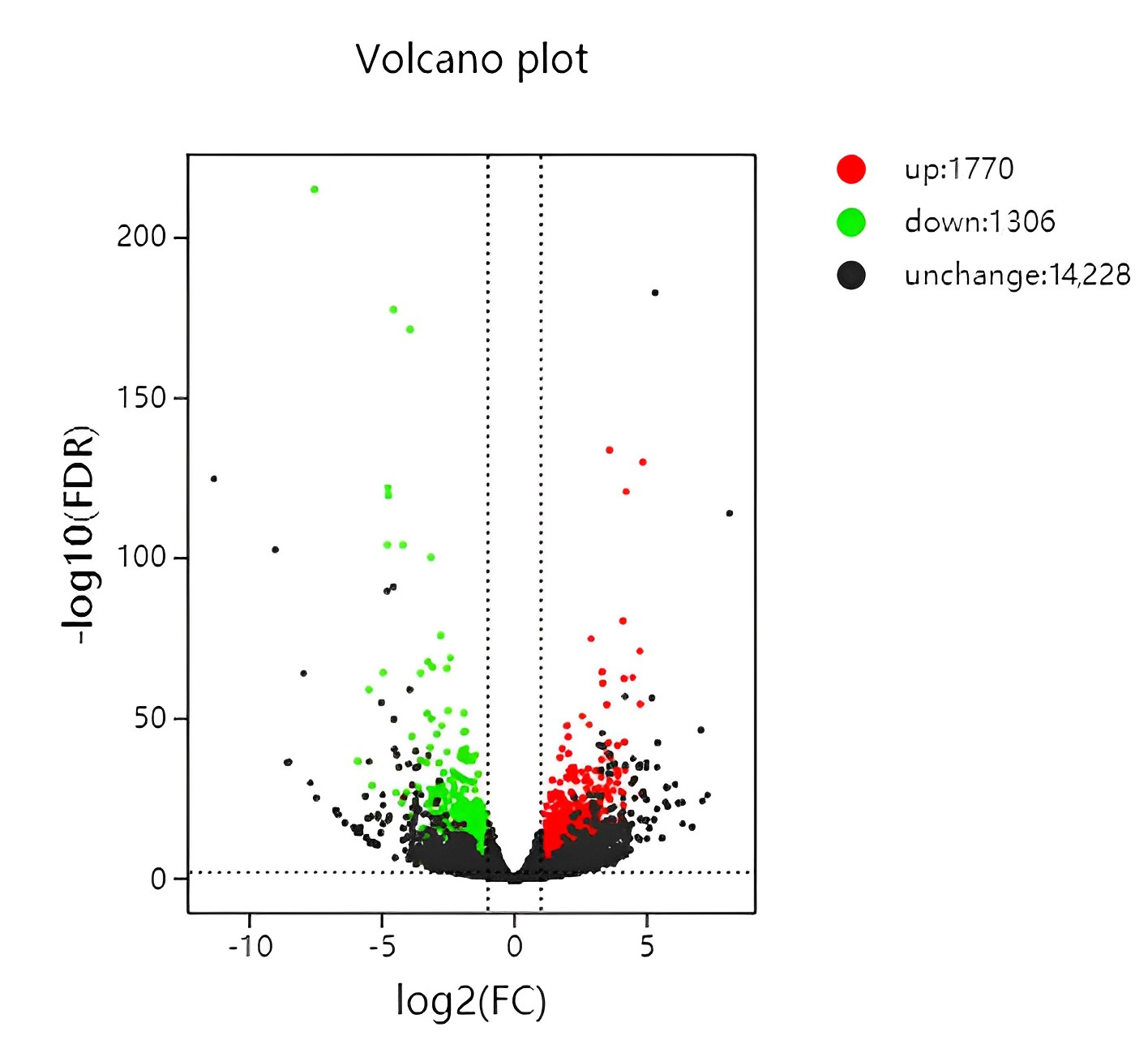

Supplement: Supplementary file 1 [file ijms-24-05737-s001.zip › Supplemental Figure S1.jpg]

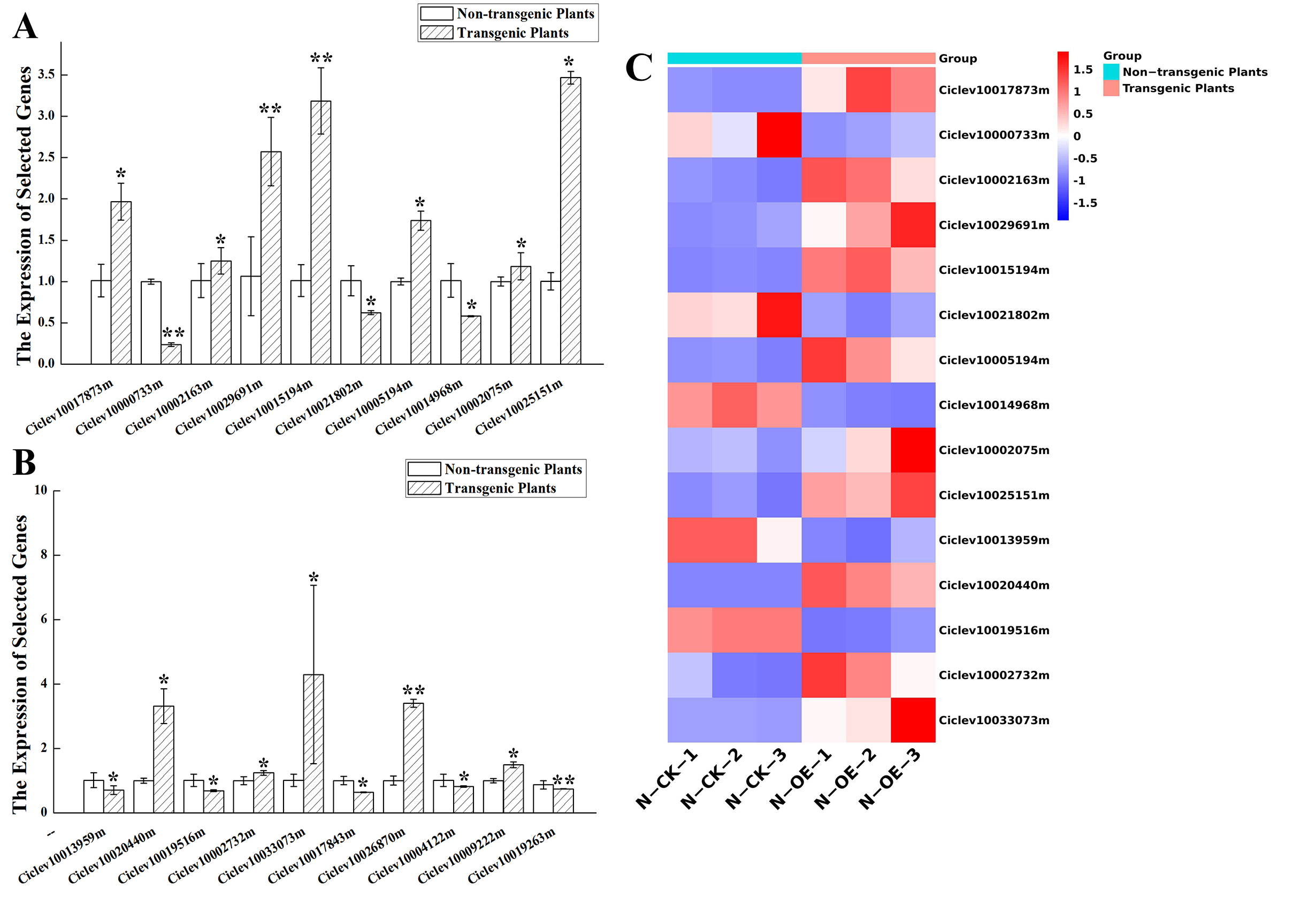

Supplement: Supplementary file 1 [file ijms-24-05737-s001.zip › Supplemental Figure S2.jpg]
